# Socioeconomic Inequalities in Non-Communicable Diseases Prevalence in India: Disparities between Self-Reported Diagnoses and Standardized Measures

**DOI:** 10.1371/journal.pone.0068219

**Published:** 2013-07-15

**Authors:** Sukumar Vellakkal, S. V. Subramanian, Christopher Millett, Sanjay Basu, David Stuckler, Shah Ebrahim

**Affiliations:** 1 South Asia Network for Chronic Diseases, Public Health Foundation of India, New Delhi, India; 2 Department of Society, Human Development and Health, Harvard School of Public Health, Harvard University, Boston, Massachusetts, United States of America; 3 Department of Primary Care and Public Health, Imperial College London, London, United Kingdom; 4 Prevention Research Center, Stanford University, Stanford, California, United States of America; 5 Department of Public Health and Policy, London School of Hygiene and Tropical Medicine, London, United Kingdom; 6 Department of Sociology, Oxford University, Oxford, United Kingdom; 7 Department of Non-Communicable Disease Epidemiology, London School of Hygiene and Tropical Medicine, London, United Kingdom; Indiana University, United States of America

## Abstract

**Background:**

Whether non-communicable diseases (NCDs) are diseases of poverty or affluence in low-and-middle income countries has been vigorously debated. Most analyses of NCDs have used self-reported data, which is biased by differential access to healthcare services between groups of different socioeconomic status (SES). We sought to compare self-reported diagnoses versus standardised measures of NCD prevalence across SES groups in India.

**Methods:**

We calculated age-adjusted prevalence rates of common NCDs from the Study on Global Ageing and Adult Health, a nationally representative cross-sectional survey. We compared self-reported diagnoses to standardized measures of disease for five NCDs. We calculated wealth-related and education-related disparities in NCD prevalence by calculating concentration index (C), which ranges from −1 to +1 (concentration of disease among lower and higher SES groups, respectively).

**Findings:**

NCD prevalence was higher (range 5.2 to 19.1%) for standardised measures than self-reported diagnoses (range 3.1 to 9.4%). Several NCDs were particularly concentrated among higher SES groups according to self-reported diagnoses (C_srd_) but were concentrated either among lower SES groups or showed no strong socioeconomic gradient using standardized measures (C_sm_): age-standardised wealth-related C: angina *C_srd_ 0.02 vs. C_sm_* −*0.17;* asthma and lung diseases *C_srd_* −*0.05 vs. C_sm_* −*0.04 (*age-standardised education-related *C_srd_ 0.04 vs. C_sm_* −*0.05);* vision problems *C_srd_ 0.07 vs. C_sm_* −*0.05;* depression *C_srd_ 0.07 vs. C_sm_* −*0.13*. Indicating similar trends of standardized measures detecting more cases among low SES, concentration of hypertension declined among higher SES (C_srd_ 0.19 vs. C_sm_ 0.03).

**Conclusions:**

The socio-economic patterning of NCD prevalence differs markedly when assessed by standardized criteria versus self-reported diagnoses. NCDs in India are not necessarily diseases of affluence but also of poverty, indicating likely under-diagnosis and under-reporting of diseases among the poor. Standardized measures should be used, wherever feasible, to estimate the true prevalence of NCDs.

## Introduction

Non-communicable disease (NCDs) are increasingly dominating health care needs in low and middle income countries (LMICs) with their importance gaining increased policy recognition over the past decade [1], [2], [3], [4]. NCDs such as heart disease, stroke, diabetes, cancer and chronic respiratory diseases are by far the leading causes of mortality representing 60% of all deaths globally - with 80% occurring in LMICs [5], [6], [7], [8].

Among LMICs, India is considered a particularly important nation to study the emerging burden of NCDs. India is projected to experience more deaths from NCDs than any other country over the next decade, due to the size of population and worsening risk factor profile, associated with recent dramatic economic growth [9], [10], [11], [12], [13], [14]. The country has deep and entrenched social and economic disparities, with affordable healthcare being beyond the reach of large sections of society [15], [16], [17]. Further, India has emerging data sources with which to study NCD risk factors [18], [19] and can serve as a policy leader on NCD control for other LMICs [20], [21].

The epidemiologic transition from a predominance of infectious diseases to NCDs is thought to arise initially in the better-off sections of a population due to their more rapid acquisition of life-styles related to economic development [22]. For example, while coronary heart disease mortality in England and Wales was initially concentrated among the wealthy, this pattern apparently reversed over two to three decades [23], although the veracity of these data is contested [24].

The epidemiological evidence on the socioeconomic status (SES) related patterning of NCDs remains limited in LMICs. Indian data drawn from the National Sample Survey Office (NSSO) 2004 found that prevalence of NCDs was highest among higher-income groups when based on self reported statistics [25]. The positive association between income and the prevalence of disease at the national level was also observed in the self-reported diabetes from the National Family Health Survey-3 [26]. Conversely, evidence from a study in Chennai, which used biochemical measures for diagnosis, revealed the prevalence of diabetes and cardio-metabolic risk factors rapidly increased in low income groups over a ten year period, such that they ‘caught up’ to those of middle income groups [27]. Furthermore, a recent study using more objective indicators confirmed greater prevalence of cardiovascular diseases risk factors among the low SES groups in India [28]. An important question is whether these contrasting findings are due to artefact, largely arising from different measurement approaches. And, are reported socioeconomic inequalities in NCDs biased by differential access to healthcare services between groups of different SES in India?

Epidemiological studies of NCDs using self-reported measures might underestimate prevalence in low SES groups as wealthier groups have relatively more access to healthcare in poor countries. More specifically, detection biases can significantly affect the rate of diagnosis between SES groups as socially disadvantaged individuals with less education and living in places with poor medical and health facilities fail to perceive and report the presence of illness and thus fail to seek healthcare, in addition to several organizational and social or cultural and financial barriers that limit the access to healthcare services [29], [30], [31], [32]. Thus, use of more standardized measures may better estimate the true prevalence of NCDs.

Here, we use Indian data from the Study on Global Ageing and Adult Health to assess socio-economic differences in NCD prevalence. We developed a standardized measure of NCD prevalence by utilizing various standardized criteria available and then compared standardised diagnostic measures (hereafter termed standardized measures) with self-reported diagnoses to examine the extent to which socio-economic inequality in NCD prevalence documented in previous studies may be due to artefact and differential access to healthcare services between groups of different SES.

## Data and Methods

We used individual level, cross-sectional data (Wave 1: version 1.1.0) from the Study on Global AGEing and Adult Health (SAGE), initiated by the World Health Organisation (WHO) (hereafter WHO SAGE survey) and conducted in India during April-August 2007. The survey was designed to generate a nationally representative sample, thus, the states selected covered diverse geographic locations and varying levels of development in India [19], [33], [34], [35] and comprised of Maharashtra (west), Karnataka (south), West Bengal (east), Assam (north east), Rajasthan (north) and Uttar Pradesh (central). Sampling methods were based on the design developed for the World Health Survey, 2002 [33] where a probability sampling strategy was employed using multi-stage, stratified, random cluster samples. The primary sampling units were stratified by region and location (urban/rural) and, within each stratum, enumeration areas were selected. Details are available on the SAGE website (www.who.int/healthinfo/systems/sage).

The households selected in a state were distributed among its rural and urban areas in proportion to their state population. The WHO SAGE aimed to generate nationally representative samples of persons aged 50+ years with comparison samples of younger adults aged 18–49 years. Only one individual aged 18–49 years was invited to complete the individual interview per household, whereas all individuals aged 50+ were invited. A total of 10424 households were surveyed with information being collected on individual health modules from 12198 individual respondents giving an overall household level response rate of 87% and an individual level response rate of 65% [35]. Of these, 5048 and 7150 individuals were aged 18–49 years and 50+ years, respectively. A weighting scheme was devised to construct a sample with nationally-representative characteristics by including sample selection and a post-stratification factor [35]. The survey instrument was conducted using an interviewer-administered questionnaire in the native language of the respondent using local, commonly understood terms. A total of five languages with back translation to English were used in the survey to ensure accuracy and comparability. Proxy respondents were identified for selected individuals who were unable to complete the interview.

Ethical clearance was obtained from research review boards local to each participating SAGE site (several of which are linked to universities), in addition to the WHO Ethical Review Committee. Informed consent was obtained from each respondent prior to interview.

### Major NCDs

Five major NCDs (under two broad categories) were identified using respondent self-reported diagnoses and standardized measures: i) Cardiovascular diseases: angina, and hypertension, and ii) Non- cardiovascular- diseases: chronic lung diseases (emphysema, bronchitis, chronic obstructive pulmonary disease) and asthma, vision problems and depression (mental disorders). The measure of both self-report of diagnoses and standardized measures of these NCDs were used.

Table 1 shows the descriptions of the relevant survey questions for self-reports of diagnosed cases as well as the criteria used for developing standardized measures for the identification of the NCDs. For self-reported diagnosed cases, participants were asked whether they had ever been diagnosed with each condition. This measure would estimate prevalence of specific NCD among only those who i) perceived presence of NCDs or their symptoms (either by people themselves or through family members or other members of society etc), and ii) reported such cases for diagnoses. At the same time, this measure excludes those cases that were undiagnosed irrespective of whether or not people had perceived the presence of NCDs.

**Table 1:** Description of the methods of both self-reported diagnoses and standardized measures for specific NCDs.

NCDs	Self-reported diagnoses (Survey Questions)	Standardized measurement
Angina	Have you ever been diagnosed with angina or angina pectoris (a heart disease)?	Each of the following 3 conditions was to be met: 1) During the last 12 months, the respondents have had experienced any pain or discomfort in the chest when walking uphill or hurry and/or ordinary pace on level ground. 2) The pain or discomfort had been relieved while just simply stopping the walk.3) The pain or discomfort site is either ‘sternum’ (all level) or ‘left anterior chest and left arm’. To identify location of pain or discomfort, respondents were asked to choose from a picture depicting numbered panels of the upper body.
Hypertension	Have you ever been diagnosed with high blood pressure (hypertension)?	Blood pressure measurement were taken with ‘Boso Medistar Wrist Blood Pressure Monitor Model S’ where the systolic blood pressure, diastolic blood pressure and pulse rate were documented. Blood pressure measurements were done three times for each respondent and at least 1 minute interval has been given between each blood pressure reading. Based on the WHO criteria of hypertension for adults 18 years and older, we defined people with hypertension as those who have reported the systolic blood pressure ≥140 mmHg and/or diastolic blood pressure ≥90 mmHg. S there were three blood pressure readings, we have taken an average of the three readings, separately for systolic blood pressure and diastolic blood pressure [41], [42].
Asthma &Chronic lungdiseases	Have you ever been diagnosed with asthma (an allergic respiratory disease)? Have you ever been diagnosed with chronic lung disease (emphysema, bronchitis, COPD)?	Spirometry was performed after the administration of an adequate dose of short-acting inhaled bronchodilator in order to minimise variability. The respondents were asked to take a deep breath and then to blow as long and hard as he/she can into a small tube attached to the spirometry machine and then FEV1 (Forced Expiratory Volume in One Second) and FVC (Forced Vital Capacity) were documented. Three trails of spirometry test were performed. The ratio of FEV_1_ (Forced Expiratory Volume in One Second) to FVC (Forced Vital Capacity) was calculated. Following the specific spirometry cut-points suggested by GOLD, those with FEV_1_/FVC <70% and also had an FEV_1_< ‘80% predicted’, in all the three trials, were termed as people with moderate and above forms of (including severe and very severe) obstructive (lung) diseases [43].
Vision problems	In the last 5 years, were you diagnosed with a cataract in one or both of your eyes (cloudiness in the lens of the eye)?	Using the four meter distance vision Tumbling E LogMAR chart, vision tests were conducted for distance vision for both left and right eyes and the recorded the resulting ‘DECIMAL’ value of each eye. If the respondents use the glasses or contact lenses, the tests were conducted using them. In accordance with WHO criteria [44], [45], we applied the definition of the low vision as an approximate Log MAR equivalent of ‘0.5 and above’ which corresponded to a ‘DECIMAL’ value of ‘0.32 and below’, thus, in our analysis, those who score ‘DECIMAL’ value of ‘0.32 and below’ in the distance vision test for at least one eye were categorized as suffering from vision problems.
Depression	Have you ever been diagnosed with depression?	In order to categorize a person as suffering from ‘moderate depression’, firstly, the following two ‘General criteria’ (i.e., General criteria 1 and 2) should be met [46]. 1) General criteria 1: At least two of the following three symptoms (conditions) must be present: i) during the last 12 months, have had a period lasting several days when felt sad, empty or depressed; ii) during the last 12 months, have had a period lasting several days when lost interest in most things usually enjoy such as personal relationships, work or hobbies/recreation, iii) during the last 12 months, have had a period lasting several days when have been feeling energy decreased or that are tired all the time. 2) General criteria 2: The depressive episode should last for at least 2 weeks. Secondly, if the above stated conditions are met, then a total of at least six conditions (symptoms) should be present to term a person as having ‘moderate depression’. These six conditions can be from the below stated three conditions from ‘General criteria 1′, and/or, from the following ‘Seven Sub-conditions’ 3) Seven Sub-conditions: i) During this period, person did feel negative about him/herself or like he/she had lost confidence and/or did frequently feel hopeless - that there was no way to improve things, ii) During this period, person did feel anxious and worried most days, iii) During this period, the person did think of death, or wish he/she were dead, and/or did he/she ever try to end own life, iv) During this period, did he/she have any difficulties concentrating; for example, listening to others, working, watching TV, listening to the radio, and/or did notice any slowing down in his/her thinking, v) He/she did notice any slowing down in his/her moving around, and/or he/she were so restless or jittery nearly every day that he/she paced up and down and couldn’t sit still, vi) He/she did notice any problems falling asleep, and/or, he/she did notice any problems waking up too early, and vii) During this period, he/she did lose his/her appetite.

Source: Author’s compilation from various sources.

Standardized measures for angina was derived from WHO-Rose angina questionnaire [36]. The WHO-Rose angina questionnaire has been widely used in epidemiological studies and some studies found that using a shortened version of the WHO-Rose angina questionnaire is adequate [37], [38]. The WHO-Rose angina questionnaire has been validated among south Asian population [39]. Recently, the full version of questionnaire has been validated among the Bangladesh population (who have similar socioeconomic and cultural characteristics as of Indian population) by comparing with cardiologists’ diagnoses and found that the WHO-Rose angina questionnaire had 53% sensitivity and 89% specificity [40]. The standardised measure used for hypertension was blood pressure measurement and then we used the systolic blood pressure and diastolic blood pressure cut-offs per WHO criteria for hypertension in adults 18 years and older [41], [42]. Results from spirometry (lung function) test was used as standardised measure for asthma and lung diseases and we followed the criteria suggested by the Global Initiative for Obstructive Lung Disease (GOLD) for identifying obstructive diseases that would include asthma, COPD, chronic bronchitis and emphysema [43]. Prevalence of vision problems were estimated using the Tumbling E LogMAR chart [44], [45]. Finally, standard criteria of ‘moderate depression’ was derived from the ICD-10 classification of mental and behavioural disorders [46].

Socio-economic and demographic variables used in our analysis include: age, gender, education, caste/tribe, geographical location and economic status. Except age, all the other included variables were defined as categorical (dummy) variables and the classification are as follows: *Gender* as male and female; *Education* as five categories: ‘No formal education’, ‘Less than primary school’ ‘Primary school completed’, ‘High/secondary school’, and ‘College/university education’, *Caste and tribe* as Scheduled caste (SC)/scheduled tribes (ST), No caste/tribe, and Other caste/tribe; *Location* as census defined urban or rural; *Economic status* as asset score quintiles: (first (lowest), and fifth (highest) quintile). A validated asset (wealth) score index, as originally reported in WHO SAGE data set [34], was derived using WHO standard approach to estimating permanent income from survey data on household ownership of durable goods, neighbourhood and dwelling characteristics, and access to water, sanitation, electricity [47].

### Statistical Analysis

Age-adjusted prevalence rates were calculated for self-reported diagnoses and standardized measures of diseases using an age weighting. We used the concentration index of inequality to quantify the magnitude of socio-economic disparities in NCD prevalence between groups. A variety of methods, ranging from simplest to sophisticated, are available to measure the socio-economic inequalities in health, including range, Gini coefficient (and associated Lorenz curve), Pseudo Gini coefficient (and associated pseudo Lorenz curve), index of dissimilarity, slope index of inequality (and the relative index of inequality- a derived measure from the slope index of inequality), and concentration index [48], [49]. The measure of range, which considers the differences between the top and bottom socioeconomic groups is considered as the simplest inequality measure reflecting the socio-economic dimension to inequalities in health, however, it does not reflect the population distribution and is not sensitive to changes in the distribution of the population across socioeconomic groups. On the other hand, the Gini coefficient, the pseudo-Gini coefficient and the index of dissimilarity reflect population distribution and sensitive to changes in the distribution of the of the population across socioeconomic groups but do not reflect the socio-economic dimension to inequalities in health [49]. The concentration index, a generalisation of the Gini coefficient [50], and the slope index (and relative index) of inequality are likely to present an accurate picture of economic inequalities in health by reflecting both the socioeconomic dimension to inequalities in health and the experiences of the entire population, and also being sensitive to changes in the distribution of the population across socioeconomic groups [49], [51], [52], [53]. There is little to choose between the concentration index and slope index (and relative index) of inequality because the slope index is equal to the generalized concentration index divided by the variance of the relative rank variable (relative rank in the socioeconomic distribution) and the relative index of inequality is equal to the concentration index divided by twice the variance of the relative rank [49]. Recently, the concentration index has been increasingly used to characterize socio-economic disparities in health in an objective manner [51], [52], [53], [54], [55], [56].

Concentration index (C) was computed as twice the (weighted) covariance of the health variable (‘ill-health’ in the present study) and a person’s relative rank in terms of economic status, divided by the variable mean, according to equation below [53], [57], [58].
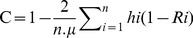
(1)where 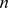
is the sample size, h_i_ is the ill-health of the i^th^ individual, µ is the (weighted) mean of the ill-health, R_i_ is the fractional rank of the i^th^ individual in terms of the index of household economic status. The value of the concentration index can vary between −1 and +1. However, when the health variable whose inequality is being investigated is binary, the minimum and maximum possible values of the concentration index are equal to *μ*−1 and 1−*μ,* respectively, where *μ* is the mean of the variable in question [59]. A negative value implies that a variable is concentrated among the lower SES while the opposite is true (i.e. concentrated among the better-off) for positive values. When there is no inequality, the concentration index will be zero [49].

Several measures of SES are available but no measure is considered as the gold standard. Standard economic measures of SES use monetary information, such as income or consumption expenditure. However, the collection of accurate income and expenditure data is a demanding task and have limitations that that, in some instances, measuring income can be difficult for the self or transitory employed (e.g. agricultural work), due to accounting issues and seasonality, people may have income and expenditure in kind, and there is possibility of under-reporting of income due to the fear to potential taxation or exclusion from social security programs, [60], [61], [62]. Education also has been used another measure of SES [48]. Recently, asset based measures that capture living standards, such as household ownership of durable assets (e.g. TV, car) and infrastructure and housing characteristics (e.g. source of water, sanitation facility) are increasingly being used to measure the SES [63], [64]. In the present study, we used wealth (asset score index) and education as two distinct indicators of SES [48], and thus estimated wealth-related concentration index and education-related concentration index in the prevalence of NCDs. The concentration index was estimated with ADePT software that was developed by the World Bank [65]. All other statistical estimations were done with the STATA version 10 (Stata Corp, College Station, Texas) [66].

## Results

### Prevalence Rate Differences between Self-reports and Standardized Measures

**Figure 1:**
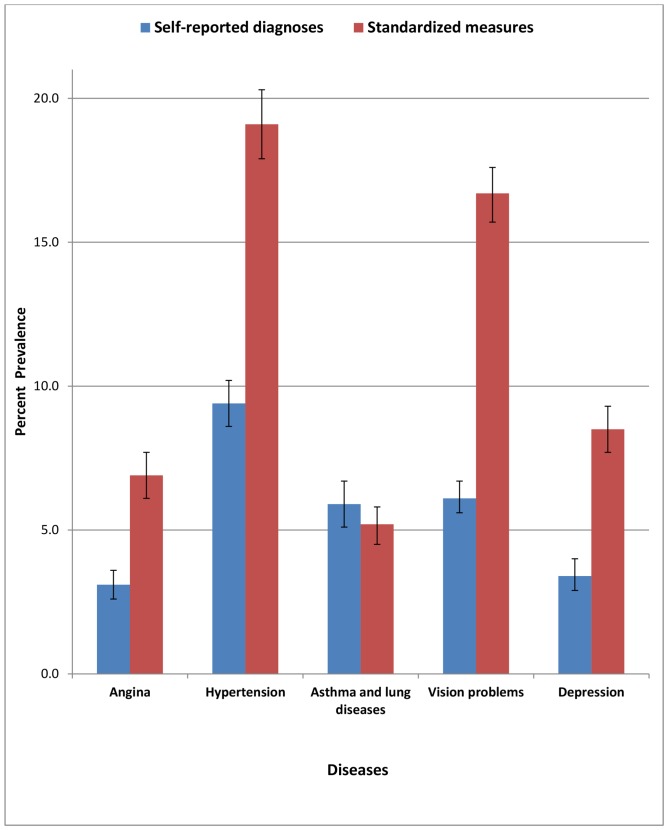
Percentage prevalence rate (with 95% CI) for self-reported diagnoses and standardized measure of diseases, among adult Indian population.

We found significantly lower prevalence rates for all NCDs when a diagnosis was based on self-report of diagnosed cases rather than standardized measures, except asthma and chronic lung diseases (Figure 1). The prevalence rate of hypertension was 9.4% according to self-report of diagnosed cases versus 19.1% using standardized measures. Similarly, the prevalence of angina was 3.1% versus 6.9%, asthma and chronic lung disease prevalence was 5.9% versus 5.2%, vision problems prevalence was 6.1% versus 16.7% and moderate depression prevalence was 3.4% versus 8.5%.

### Disparities in NCD Prevalence by Wealth and Education

**Figure 2:**
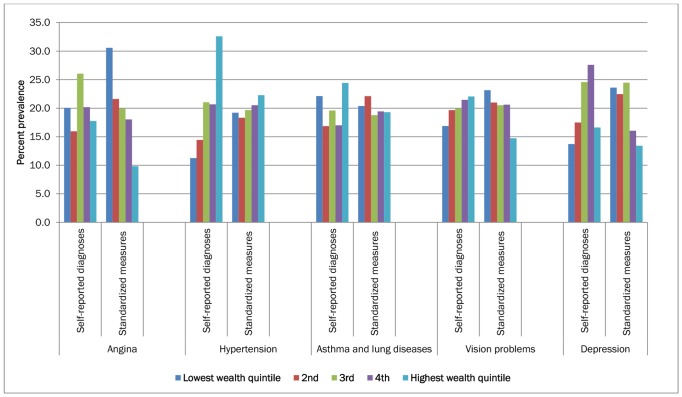
Percentage distribution of age-standardized prevalence rate of self-reported diagnoses and standardized measure of diseases among adult Indian population in 2007, by income quintiles.

**Figure 3:**
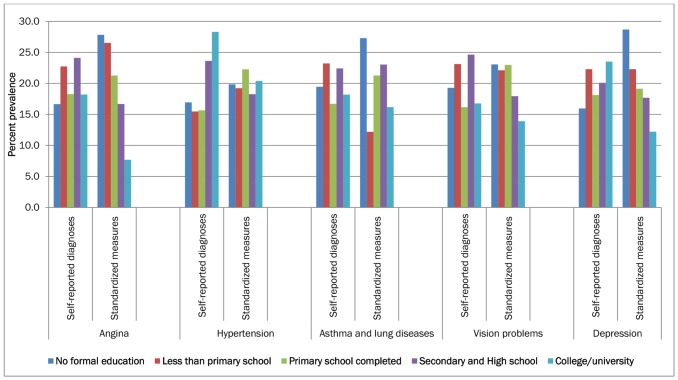
Percentage distribution of age-standardized prevalence rate of self-reported diagnoses and standardized measures of diseases among adult Indian population in 2007, by education groups.

The age-standardised prevalence rate of the diseases, using both self-report of diagnosed cases and standardized measures, by demographic and socio-economic variables are presented in Table 2 and Figures 2 and 3. Self-reported diagnosed cases of disease prevalence were significantly higher in the most affluent quintile compared with the least affluent quintile. Conversely, disease prevalence measured using standardized measures tended to show either negative or no strong association with wealth. Similar trends were observed for educational level too.

**Table 2:** Age-adjusted prevalence rate (in %, with 95% CI) of disease measured through self-reported diagnoses and standardized measures criteria, by socio- economic characteristics, among adult Indians in 2007.

		Angina		Hypertension		Asthma and lung diseases		Vision problems		Depression	
Socio-economic characteristics		Self-reported diagnoses	Standardized measures	Self-reported diagnoses	Standardized measures	Self-reported diagnoses	Standardized measures	Self-reported diagnoses	Standardized measures	Self-reported diagnoses	Standardized measures
**Sex**	Female	3.0(2.5;3.6)	8.1(7.2;9.1)	12.4(11.3;13.5)	19.5(18.1;20.8)	4.1(3.4;4.8)	5.6(4.8;6.4)	6.5(5.9;7.2)	19.6(18.4;20.8)	2.6(1.9;3.2)	10.0(9.0;11.0)
	Male	3.2(2.4;4.0)	6.1(4.8;7.5)	7.0(5.8;8.2)	19.3(17.3;21.4)	7.2(6.0;8.5)	5.0(3.9;6.1)	5.7(4.9;6.5)	14.3(13;15.7)	4.1(3.2;5.1)	7.5(6.0;8.9)
**Location**	Rural	2.8(2.3;3.3)	7.2(6.4;8.0)	8.3(7.4;9.1)	18.2(17;19.5)	5.8(5.0;6.7)	5.5(4.8;6.2)	6.0(5.5;6.6)	17.3(16.3;18.2)	3.5(2.9;4.1)	8.5(7.7;9.4)
	Urban	3.8(2.6;5.1)	5.9(4.4;7.5)	12.0(10.2;13.9)	22.4(19.5;25.3)	5.5(4.0;6.9)	4.6(3.2;6.1)	6.2(4.9;7.5)	15.4(13.3;17.5)	2.8(1.8;3.9)	8.4(6.7;10.1)
**Caste/Tribe**	Scheduled caste/tribe	2.6(1.7; 3.5)	7.7(6.2; 9.2)	6.7(5.5; 8.0)	20.6(18.3; 22.9)	5.4(4.5; 6.3)	5.0(3.8; 6.2)	5.3(4.4; 6.2)	15.7(14.2; 17.2)	3.1(2.2; 4.1)	7.4(6.2; 8.6)
	No caste/tribe	4.5(3.4; 5.6)	7.3(5.4; 9.1)	9.1(7.5; 10.8)	21.7(19.0; 24.4)	7.1(5.3; 8.8)	3.4(2.2; 4.7)	8.1(6.7; 9.5)	17.2(15.1; 19.4)	10.1(7.9; 12.4)	12.1(10.1; 14.2)
	Other caste/tribe	3.0(2.3; 3.7)	6.6(5.6; 7.6)	10.6(9.4; 11.7)	17.9(16.4; 19.4)	5.8(5.0; 6.6)	5.7(4.8; 6.6)	5.9(5.2; 6.6)	17.2(15.9; 18.4)	2.0(1.4; 2.6)	8.4(7.3; 9.5)
**Education**	No formal education	2.7(1.9;3.4)	8.5(7.2;9.8)	8.6(7.4;9.8)	19.3(17.2;21.3)	5.1(4.2;6.0)	6.3(5.0;7.5)	5.7(5.0;6.4)	18.0(16.7;19.3)	2.6(1.9;3.3)	10.5(9.1;11.9)
	Less than primary school	3.6(2.3;4.9)	8.1(6.0;10.1)	7.9(6.0;9.7)	18.7(15.3;22.0)	6.7(4.5;8.7)	2.8(1.9;3.7)	6.9(5.4;8.4)	17.3(14.5;20.1)	3.7(2.1;5.2)	8.2(6.1;10.3)
	Primary school completed	2.9(2.0;3.8)	6.5(4.8;8.1)	8.0(6.5;9.7)	21.6(18.8;24.4)	5.3(3.8;6.8)	4.9(3.6;6.2)	4.8(3.9;5.7)	17.9(15.5;20.4)	3.0(1.9;4.1)	7.1(5.5;8.9)
	Secondary and High school	3.8(2.7;4.9)	5.1(3.7;6.4)	12(10.1;13.9)	17.7(15.5;19.9)	6.5(4.8;8.1)	5.3(4.0;6.6)	7.3(6;8.7)	14(12.3;15.7)	3.3(2.3;4.3)	6.4(5.0;7.8)
	College/university	2.9(1.3;4.5)	2.3(0.6;4.0)	14.4(11.5;17.3)	19.8(16.0;23.5)	4.7(2.4;7.1)	3.7(2.2;5.3)	5.0(3.4;6.6)	10.9(8.4;13.3)	3.9(2.1;5.7)	4.8(1.8;7.8)
**Wealth (asset) Quintile**	Lowest quintile	3.0(2.0;4.0)	10.7(8.7;12.7)	5.3(3.9;6.6)	18.4(15.8;21)	7.5(5.7;9.3)	5.3(3.9;6.7)	5.1(4.1;6.1)	19.5(17.2;21.7)	2.4(1.4;3.3)	10.1(8.3;12.0)
	2nd	2.4(1.5;3.3)	7.6(5.9;9.2)	6.7(5.4;8.1)	17.5(15.2;19.9)	5.0(3.7;6.3)	5.7(4.4;7.0)	5.9(4.8;7.1)	17.6(15.6;19.7)	3.0(1.8;4.2)	9.5(7.9;11.1)
	3rd	3.9(2.6;5.2)	7.0(5.5;8.4)	9.8(7.9;11.7)	18.8(16.5;21.2)	5.9(4.3;7.4)	4.9(3.5;6.2)	6.0(4.9;7.2)	17.2(15.3;19.2)	4.2(2.9;5.7)	10.2(8.5;12.0)
	4th	3.0(2.0;4.0)	6.3(4.7;7.9)	9.7(8.1;11.3)	19.7(17.2;22.1)	5.2(3.7;7.0)	5.0(3.7;6.3)	6.5(5.2;7.7)	17.3(15.3;19.3)	4.8(3.4;6.2)	6.8(5.3;8.2)
	Highest quintile	2.7(1.9;3.4)	3.4(2.4;4.4)	15.2(13;17.4)	21.3(18.5;24.2)	5.6(3.7;7.5)	5.0(3.7;6.3)	6.6(5.5;7.7)	12.4(10.9;13.9)	2.9(1.8;3.9)	5.6(4.1;7.0)
	**Overall**	3.1(2.6;3.6)	6.9(6.1;7.7)	9.4(8.6;10.2)	19.1(17.9;20.3)	5.8(5.0;6.6)	5.2(4.6;5.9)	6.1(5.5;6.6)	16.7(15.8;17.7)	3.4(2.8;3.9)	8.5(7.7;9.3)

Notes: i) Figures in the parentheses show 95% confidence intervals, ii) Source: Authors estimate from WHO SAGE survey, 2007.

Table 3 shows the wealth-related and the education-related concentration indices (C) of the diseases in terms of i) Unstandardized C, ii) Age-standardised C, and iii) Age and sex standardized C, separately for self-report of diagnosed cases (C_srd_) and standardized measures (C_sm_).

**Table 3:** Wealth-related concentration index of the prevalence of the diseases measured through self-reported diagnoses and standardized measures, among adult Indians in 2007.

	Unstandardized C		Age-standardized C		Age and sex standardized C	
Diseases	Self-reported diagnoses(C_srd_)	Standardized measures(C_sm_)	Self-reported diagnoses(C_srd_)	Standardized measures(C_sm_)	Self-reported diagnoses(C_srd_)	Standardized measures(C_sm_)
**Wealth-related concentration index**						
Angina	0.02(−0.06; 0.11)	−0.19(−0.25; −0.12)	0.02(0.01; 0.02)	−0.17(−0.18; −0.16)	0.01(0.01; 0.02)	−0.17(−0.17; −0.16)
Hypertension	0.21(0.16; 0.26)	0.04(0.00; 0.07)	0.19(0.18; 0.19)	0.03(0.03; 0.04)	0.19(0.18; 0.19)	0.03(0.03; 0.04)
Asthma and lung diseases	0.03(−0.08; 0.13)	−0.04(−0.11; 0.04)	−0.05(−0.06; −0.05)	−0.04(−0.04; −0.04)	0.01(0.01; 0.02)	−0.04(−0.04; −0.04)
Vision problems	0.08(0.03; 0.13)	−0.05(−0.08; −0.01)	0.07(0.07; 0.07)	−0.05(−0.06; −0.05)	0.07(0.07; 0.07)	−0.06(−0.06; −0.05)
Depression	0.05(−0.04; 0.14)	−0.13(−0.18; −0.08)	0.07(0.06; 0.07)	−0.13(−0.13; −0.12)	0.07(0.06; 0.07)	−0.13(−0.13; −0.12)
**Education-related concentration index**						
Angina	0.05(−0.07; 0.16)	−0.11(−0.23; 0.00)	0.03(0.03; 0.03)	−0.13(−0.13; −0.12)	0.03(0.03; 0.03)	−0.15(−0.16; −0.15)
Hypertension	0.14(0.06; 0.21)	0.03(−0.02; 0.09)	0.12(0.12; 0.13)	0.01(0.01; 0.01)	0.13(0.12; 0.13)	0.00(0.00; 0.00)
Asthma and lung diseases	0.07(−0.09; 0.23)	0.00(−0.09; 0.10)	0.04(0.04; 0.04)	−0.05(−0.06; −0.04)	0.08(0.07; 0.08)	−0.04(−0.05; −0.03)
Vision problems	−0.02(−0.10; 0.06)	−0.11(−0.17; −0.05)	0.02(0.02; 0.02)	−0.06(−0.06; −0.06)	0.01(0.01; 0.02)	−0.06(−0.06; −0.06)
Depression	0.05(−0.08;0.18)	−0.07(−0.16;0.02)	0.06(0.06;0.06)	−0.09(−0.10;−0.08)	0.06(0.06;0.06)	−0.08(−0.08; −0.07)

Several NCDs were concentrated among the lower SES groups using standardized measures whereas self-reported diagnosed cases indicated concentration among the higher SES groups, however, with considerable variation in the magnitude, beyond chance as reflected by the confidence intervals (See Table 3). Each of the two cardiovascular diseases has a higher concentration of disease among higher SES groups according to self-reported diagnoses. The age-standardised wealth-related concentration index and education-related concentration index for standardised measures of the prevalence of angina is −0.17 and −0.13, respectively. It means that the prevalence of angina (identified through standardized measures) is concentrated among the poor and the less-educated, which implies that a 17% reduction in the prevalence of angina among the poor and 13% reduction in the prevalence of angina among the less-educated would eradicate the observed disparity in the total prevalence of angina across SES groups. On the other hands, the self-reported diagnoses of angina was concentrated among the rich (wealth-related C_srd_ 0.02) and the highly-educated (education-related C_srd_ 0.03). The self-reported diagnosed cases of hypertension is highly concentrated among the higher SES and continue to concentrate among higher SES groups per standardized measures but with considerable attenuation as standardized measures detect relatively more cases of hypertension among low SES (wealth-related *C_srd_ 0.19 vs. C_sm_ 0.03 and education-related C_srd_ 0.12 vs. C_sm_ 0.01)*.

The age-standardised concentration index for self-reported diagnoses of asthma and lung diseases (wealth-related *Csrd* −*0.05 vs. Csm* −*0.04;* education-related *Csrd 0.04 vs. Csm* −*0.05),* vision problems (wealth-related *Csrd 0.07 vs. Csm* −*0.05;* education-related *Csrd 0.02 vs. Csm* −*0.06)* and depression (wealth-related *Csrd 0.07 vs. Csm* −*0.13;* education-related *Csrd 0.06 vs. Csm* −*0.09)* showed positive values of C (i.e. concentration among the affluent/better educated), whereas negative values of C were found for standardized measures (i.e. concentration among the poor/less educated), with exception of asthma and chronic lung diseases showing negative value for wealth-related *Csrd* but positive value for education-related *Csrd*.

**Figure 4:**
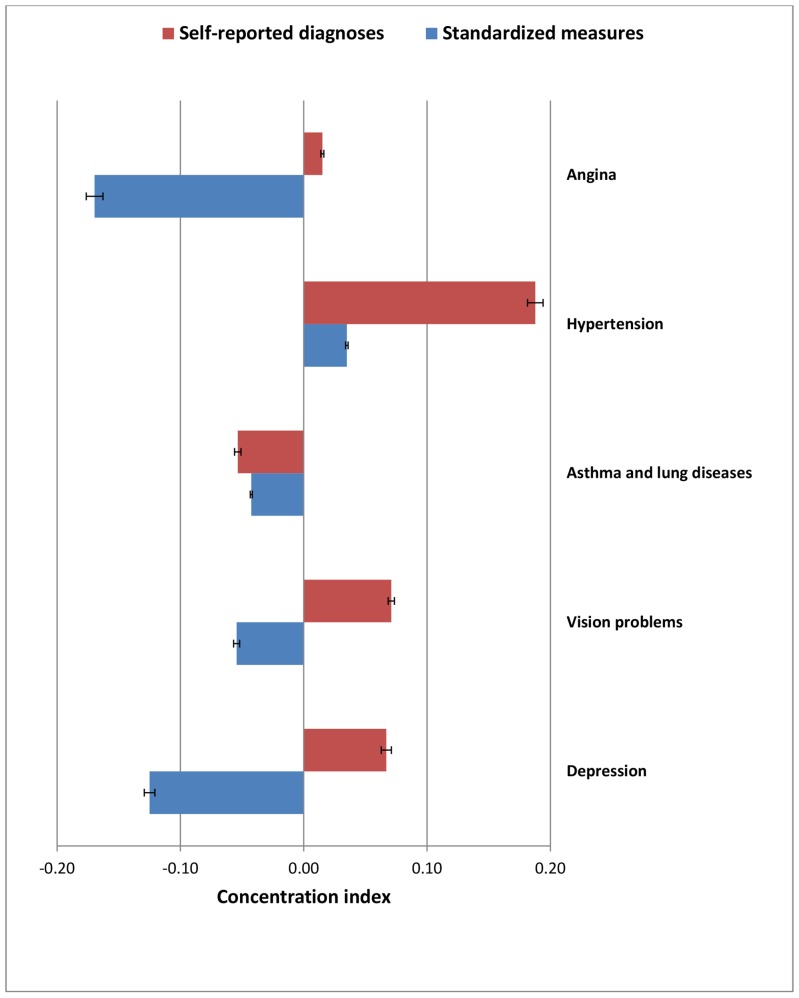
Age-standardized wealth-related concentration index (with 95% CI) for self-reported diagnoses and standardized measures of diseases, among adult Indian population.

**Figure 5:**
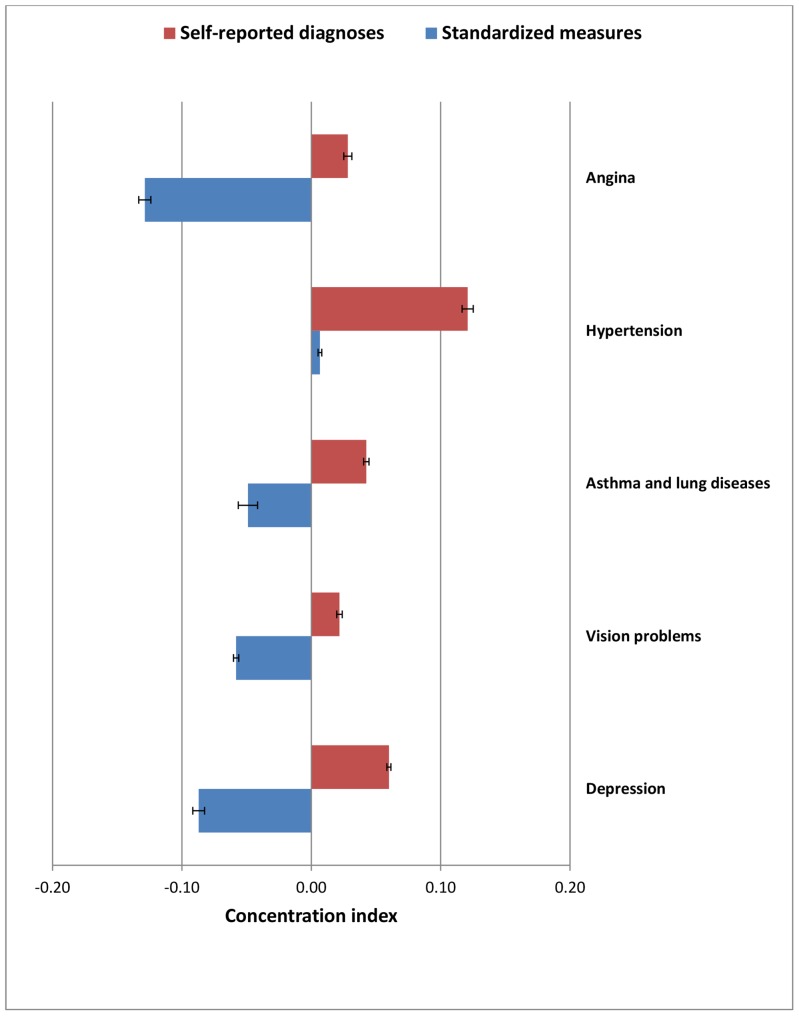
Age-standardized education-related concentration index (with 95% CI) for self-reported diagnoses and standardized measures of diseases, among adult Indian population.

As shown in Figure 4 and Figure 5, the majority of NCDs were concentrated among higher SES groups using self-reports but were concentrated among lower SES groups or showing no strong gradient based on standardized measures, indicating considerable probable under-diagnosis and under-reporting of diseases among lower SES groups.

## Discussion

We have demonstrated that the prevalence and socioeconomic disparities in several NCDs among the Indian adult population using nationally representative data depend on whether self-reported diagnoses or standardized measures of NCDs are used. We attempted to incorporate more standardised measures of prevalence of NCDs as self-reported diagnosed cases of NCDs might be poor indicators of the true prevalence of NCDs across various SES groups where people especially from the lower SES and remote place might often fail to perceive and report the illness, and those who were able to perceive might fail to access healthcare due to several constraints. We found that wealth-related and education-related concentration of disease varied with greater concentration among the affluent/educated when using self-reported diagnoses and either among lower SES groups or showed no strong SES gradient when using standardized measures of disease. This may be consistent with observations that NCDs are typically either under-reported or under-diagnosed in LMICs, including India [29], [67]. These findings provide salient information for the ongoing debate about whether NCDs in LMICs are concentrated among the affluent or among poorer groups as in high income countries [68]. Furthermore, our findings suggest that self-reported NCD prevalence estimates may be highly misleading if used to determine burden of disease or for targeting interventions.

Though our analysis found statistically significant values of age-standardised concentration indices for each of the five NCDs, no sweeping generalisation about the concentration of NCDs among the affluent or the poor should be made. First, the patterns vary depending on the method used to quantify prevalence of NCD. Second, the patterns vary depending on the disease considered. Third, the magnitude of concentration indices for both self-reported diagnoses and standardized measures were not very high. For instance, the education-related concentration index for the standardized measures of asthma and lung disease is −0.05, which means that a mere 5% reduction of the prevalence of asthma and lung diseases among the less-educated would eradicate the observed disparity in its total prevalence.

Previous studies in India have demonstrated the increased risk of cardiovascular disease and cardio-metabolic risk factors in affluent groups [69], [70], [71], and have highlighted the higher levels of tobacco use among poorer groups [9], [72], indicating the likelihood of increased cardiovascular disease and other NCDs in the future. Comparisons of socio-economic patterning of NCDs using self-reported diagnoses and standardized measures within the same nationally representative datasets have not previously been reported. Our study indicates that the socio-economic patterning of NCDs found in LMICs may depend on the diagnostic criteria employed. A recent study conducted in rural India [73] found lower rates of screening for elevated blood pressure, blood glucose and cholesterol in lower SES groups. This is consistent with our assertion that the lower self-reported prevalence of NCDs in low SES groups, identified here and elsewhere, may be due to under-diagnosis and under-reporting arising from poorer access to high quality health care.

There are several important caveats and limitations to this study. First, our data do not include younger people (below 18 years). However, this would have limited bearing on findings, except possibly for asthma and lung diseases, as most of the conditions studied largely occur in adults. Second, we used cross-sectional data which prevent us from making causal inferences on factors contributing to disparities in prevalence rate. We cannot capture, using such data, the full longitudinal trends that may differ between socioeconomic groups to evaluate whether an epidemiological transition is occurring. Differences in prevalence rates between self-reported diagnoses and standardized measure may vary in the future, particularly if proposals for universal health care in India are achieved [74]. Third, though this study incorporated standardized measures to identify NCDs to the extent possible, more comprehensive and reliable indicators such as biomarkers of disease and clinical examination are required to validate these results. Some of the standardized measures used in the WHO SAGE study to assess the prevalence of angina and depression, were based on symptom reports, and the reliability of such measures is also limited by lack of comprehensiveness and is also subject to reporting biases. Considerations such as health literacy are important to account for, and may bias our results toward underestimating prevalence rates among the poor, who may have a more difficult time comprehending questions about disease symptomatology. Finally, our study could not include the standardized measure for diabetes, one of the major NCDs that has a prevalence of 3.1% and concentration among the better-off (wealth-related *C_srd_ 0.24;* education-related *C_srd_ 0.25)* per self-reported diagnoses. Furthermore, stroke and arthritis (with self-reported diagnoses prevalence rate of 1.0% and 9.4%, respectively) were excluded from our analysis as the prevalence measures available from the WHO SAGE survey were not sufficient enough to construct standardised measures of prevalence.

Our study has important policy implications. As a part of the growing attention to the prevention and management of NCDs in LMICs, early detection of chronic diseases is now part of Indian national policy (National Program for Prevention and Control of Cancer, Diabetes, Cardiovascular Diseases and Stroke-NPCDCS 2010), and similar efforts are being considered in other LMICs. Similar to other NCDs, mental health had so far not been in the policy priorities in India, where scarcity in mental healthcare infrastructure and social stigma in accessing mental health are major concerns, and the findings from this study will inform policy making as the Indian government has recently been considering a national mental health bill. It is important that the potential impact of such programmes on health disparities is assessed during their design, implementation and evaluation [75]. Our findings suggest that use of self-reported diagnoses of NCDs may lead to erroneous conclusions about policy impacts and that more standardized objective diagnostic criteria for NCDs should be used wherever feasible. While policy interest in NCD prevention in LMICs is increasing, many health and development programmes still neglect NCDs on the grounds that they are diseases of affluence. Our findings add to growing evidence that this may no longer be a tenable proposition.

Further research into the mechanisms that may explain discrepancies between self-reported diagnoses and standardized or more objective measures of the prevalence of NCDs is warranted. Moreover, we need further evidence on whether transitions of diseases from affluent to poor groups occurs in LMICs, as its occurrence has been questioned in high income countries [15]. Several theories about the rise in NCDs have assumed that higher income correlates with the increased intake of energy-dense and unhealthy foods that are high in fat, sugars and total calories, as well as increased sedentary lifestyles, which is believed to relate to obesity and heart disease. This theory does not adequately explain our findings.

In summary, we found that self-reported diagnoses prevalence rates of NCDs were concentrated among the affluent while standardized measures of the same NCDs showed them to be concentrated among the poor or show no gradient. This suggests considerable problems of access to healthcare for NCD diagnosis as well as under-reporting of diseases condition among the poor. Strategies to reduce socioeconomic inequalities in NCD prevalence should be further investigated by testing hypotheses that may further explain disparities in risk, disparities in diagnosis, and effective intervention among the highest-prevalence groups. Moreover, disease-wise in-depth investigation of the socio-economic determinants of each chronic diseases is required instead of considering all NCDs in one basket. Health development programmes should consider re-orientating their programmes to include NCDs which are diseases associated with poverty and are impoverishing.
